# Endoplasmic Reticulum-Associated rht-PA Processing in CHO Cells: Influence of Mild Hypothermia and Specific Growth Rates in Batch and Chemostat Cultures

**DOI:** 10.1371/journal.pone.0144224

**Published:** 2015-12-11

**Authors:** Mauricio Vergara, Julio Berrios, Irene Martínez, Alvaro Díaz-Barrera, Cristian Acevedo, Juan G. Reyes, Ramon Gonzalez, Claudia Altamirano

**Affiliations:** 1 Institute of Chemistry, Pontificia Universidad Católica de Valparaíso, Av. Universidad 330, Curauma, Chile; 2 School of Biochemical Engineering, Pontificia Universidad Católica de Valparaíso, Av. Brasil 2085, Valparaíso, 4059, Chile; 3 Biotechnology Center “Dr. Daniel Alkalay Lowitt”, Universidad Técnica Federico Santa María, Av. España 1680, Valparaíso, Chile; 4 Department of Chemical and Biomolecular Engineering, Rice University, Houston, Texas, United States of America; 5 Department of Bioengineering, Rice University, Houston, Texas, United States of America; 6 CREAS CONICYT Regional GORE Valparaíso R0GI1004. Av. Universidad, Curauma, Chile; Duke University Medical Center, UNITED STATES

## Abstract

**Background:**

Chinese hamster ovary (CHO) cells are the main host for producing recombinant proteins with human therapeutic applications mainly because of their capability to perform proper folding and glycosylation processes. In addition, mild hypothermia is one of the main strategies for maximising the productivity of these systems. However, little information is available on the effect of culture temperature on the folding and degradation processes of recombinant proteins that takes place in the endoplasmic reticulum.

**Methods:**

In order to evaluate the effect of the mild hypothermia on processing/endoplasmatic reticulum-associated degradation (ERAD) processes, batch cultures of CHO cells producing recombinant human tissue plasminogen activator (rht-PA) were carried out at two temperatures (37°C and 33°C) and treated with specific inhibitors of glycosylation and ERAD I (Ubiquitin/Proteasome system) or ERAD II (Autophagosoma/Lisosomal system) pathways. The effect of mild hypothermia was analysed separately from its indirect effect on specific cell growth rate. To do this, chemostat cultures were carried out at the same incubation conditions as the batch cultures, controlling cell growth at high (0.017 h^-1^) and low (0.012 h^-1^) dilution rates. For a better understanding of the investigated phenomenon, cell behaviour was also analysed using principal component analysis (PCA).

**Results and Conclusion:**

Results suggest that rht-PA is susceptible to degradation by both ERAD pathways studied, revealing that processing and/or ERAD processes are sensitive to temperature cultivation in batch culture. Moreover, by isolating the effect of culture temperature from the effect of cell growth rate verifyed by using chemostat cultures, we have found that processing and/or ERAD processes are more sensitive to reduction in specific growth rate than low temperature, and that temperature reduction may have a positive effect on protein processing. Interestingly, PCA indicated that the integrated performance displayed by CHO cells is modulated predominantly by specific growth rate, indicating that the culture temperature has a lower weighted effect within the range of conditions evaluated in this work.

## Introduction

Chinese hamster ovary (CHO) cells are the main host for the production of different biopharmaceuticals, primarily due to their ability to perform the proper folding and glycosylation required for the biological function of these molecules [1]. However, this cell system presents several requirements, such as a complex nutrient culture medium, toxic by-product accumulation, and limited oxygen transfer; combined with a limited capacity of cell growth, these requirements restrict both the longevity of the cultures and the specific productivity of the recombinant protein [2,3].

To overcome some of these limitations, different approaches have been undertaken, attempting to maximise the productivity of these systems. One of the most important is the use of mild hypothermia culture condition (35°C to 30°C), which in many cases increases the longevity of cultures and the specific productivity for a wide range of recombinant proteins in batch cultures of CHO cells [4,5,6,7], though reduced temperature does not always lead to increased specific productivity [8,9], being this phenomena cell line- and product-dependent. Some possible contributing factors involved in this mild hypothermia effect are: cell cycle arrest, apoptosis delay, an increase in the amount and/or stability of r-proteins mRNA [10] and an increase in the folding capacity and expression of endoplasmic reticulum (ER) chaperones [11,12].

Using genomic and proteomic analysis, Baik et al. [11] described under conditions of mild hypothermia (33°C) a significant increase of PDI and ERp57 levels, two known chaperones of the ER, suggesting that the r-protein was processed better when applying this condition. Similarly, an increase of 25–75% in ER chaperone expression and ER size were described by Gomez et al. [13] at mild hypothermia condition, strongly suggesting that r-protein processing in the ER could be one contributing factor to the described mild hypothermia-increased r-protein productivity [14], among other possible causes for this increment, such as the cell cycle stage detention or mRNA expression and stability.

Within the past ten years, the processes such as glycosylation, folding and degradation in the ER have attracted significant attention in the field of recombinant protein production [14]. Endoplasmic reticulum-associated degradation (ERAD) consists of the proteolytic elimination of misfolded proteins in the ER. There are two well-known ERAD pathways. One is related to degradation via proteasome in the cytoplasm after translocation and ubiquitylation by the so-called ERAD Ubiquitin/Proteasome system [15,16], and the other pathway is associated with autophagosome formation, in which protein aggregates are captured from the ER and later undergo fusion with the lysosome (ERAD autofasomal/lysosome pathway)[17,18]. According to the literature and under different culture conditions, it has been suggested that the processes of transcription, transduction and those associated to post-transduction processing can be rate limiting in r-protein synthesis and production, depending on the r-protein and productivity levels of the cell line involved [19].

Thus, although the previously mentioned investigations have enhanced the understanding of the cell mechanisms involved in the synthesis and productivity of r-proteins under mild hypothermia culture conditions, the effect of mild hypothermia on post-translation cellular events remains poorly understood. Moreover, recent studies developed in chemostat cultures have revealed that the effect of mild hypothermia on specific protein production is the result of the combined effect of temperature reduction and decreased cell growth rate since these two variables can be independently manipulated in chemostat culture [20]. Thus, chemostat culture results in a strategy to control population growth in the reactor, and allow work at a specified μ, resulting in an advantage over batch cultures where u is continuously changing according to the multiple culture variables affecting population growth.

In this work, the effect of mild hypothermia and controlled cell growth rate on ERAD in rht-PA expressing CHO cells was investigated using chemostat culture. Two temperatures (33 and 37°C) and two growth rates (0.017 and 0.012 h^-1^) were established. Chemostat cultures operating at steady-state were perturbed using an inhibitor of protein glycosylation and inhibitors of either the ERAD I (ubiquitin/proteasome) or ERAD II (autophagosome/Lysosome) pathways. The concentrations of intracellular and extracellular rht-PA, were evaluated. The associations of the different variables (independent and dependent) were analysed using the mathematical technique of principal component analysis (PCA).

## Materials and Methods

### 2.1 Cell line and culture medium

The rht-PA producing cell line (CHO TF 70R) was obtained from Pharmacia & Upjohn S.A. (Sweden) (a kind gift of Torsten Björlig). The culture medium SFM4CHO (HyClone, free of glucose and glutamine) was used. This medium was supplemented with: 20 mM glucose (G7021, Sigma, USA) and 6 mM glutamate (G8415, Sigma, USA) for batch culture, and 10 mM glucose and 6 mM glutamate for continuous culture.

### 2.2 Batch cultures

Batch cultures were started in spinner flasks (Techne, UK) with a working volume of 100 ml and were agitated at 50 rpm in an incubator (Forma Scientific CO2 incubator, Thermo Fisher Scientific Inc., USA) at 95% relative humidity in an atmosphere of 5% CO_2_. The cultures were inoculated with cells from the mid-exponential phase of growth at a cell concentration of 0.8 x 10^6^ cells/ml, this empirically determined condition was used to ensure an adequate amount of viable cells after treatment with inhibitors. The culture temperatures were controlled at 33 and 37°C ± 0.1°C. The cells were treated using the inhibitors of either the ERAD I pathway: MG-132 (2.5 μM, cell membrane permeant, [21]) (474790, Merck) or the ERAD II pathway: pepstatin A (5 μM; aspartic protease inhibitor, cell membrane permeant; [22]) (516481, Merck), leupeptin (5 μM; serine, cysteine, threonine peptidase inhibitor; low cell membrane permeability but used in intact cell studies; [23]) (108976, Merck) and E64d (5 μM; thiol protease inhibitor, cell membrane permeant; [24]) (330005, Merck). These inhibitors were used together with tunicamycin (2.5 μg/ml) (TM, T7765, Sigma-Aldrich), an inhibitor of N-acetylglucosamine transferases (cell membrane permeant; [25],[26]), to increase misfolded proteins and make ERAD degradation (and its pharmacological inhibition) more determinant of intracellular rht-PA content and/or protein release to the media.

### 2.3 Chemostat cultures

Chemostat culture experiments were performed in spinner flasks (Techne, UK) while maintaining a working volume of 150 ml. The cultures were inoculated and operated in batch-mode over 48 h and were supplied with sterile feed throughout the period of operation [27].

A series of four experiments was performed in duplicates at 37°C or 33°C, by keeping D (dilution rate) at 0.017 and 0.012 h^-1^. Samples were taken every 24 h for viable cell quantification. Each sample was centrifuged, and then the supernatant was immediately frozen at -20°C for analytic measurements. Cultures were considered to reach the steady-state (SS) when, after at least four residence times, both the number of viable cells and lactate concentration were constant in two consecutive samples. Once SS was reached, the cultures were perturbed using the inhibitor of either the ERAD I or ERAD II pathway. Samples were taken at SS and at 24 and 48 h after the addition of inhibitors.

### 2.4 Analytical methods

Cells were counted using a haemocytometer (Neubauer, Germany). Cell viability was determined by the method of trypan blue exclusion (T8154, Sigma, USA) (1:1 mixture of 0.2% trypan blue in saline and cell sample). Glucose and lactate concentrations were determined with an automatic biochemistry analyzer (YSI 2700, Yellow Springs Inc., USA). The quantities of intracellular (rht-PA(i)) and extracellular rht-PA were quantified by enzyme immunoassay (Trinilize tPA-antigen kit, Tcoag Ltd., Ireland). The enzymatic activity of rht-PA was measured by amidolytic assay (S-2288 peptide, Chromogenix, Italy).

### 2.5 Estimation of specific rates for chemostat cultures

The specific growth rate (μ) was determined by a mass balance for the bioreactor. At SS, the variation of total cells is zero. Therefore,

$$\mu =D\frac{\left(N_{v}+N_{d}\right)}{N_{v}}=D\frac{N_{T}}{N_{v}}$$ (Eq 1)

Where D is the dilution rate in (1/h), N_v_ is the viable cell number in (10^6^ cells/ml), N_d_ is the dead cell number in (10^6^ cells/ml), and N_t_ is the total cell number in (10^6^ cells/ml).

In transient states, the total cell number varies with time. Therefore,

$$\frac{dN_{T}}{dt}=\mu N_{v}-DN_{T}$$ (Eq 2)

In this case, μ was calculated numerically, first by fitting a function that describes the variation in cell number over time Xv(t). Next, this function was derived and subsequently was evaluated over time.

The specific consumption/production rates of the compound of interest (q_i_) in (nmol/10^6^ cells*h) were determined from a material balance in the bioreactor under steady state conditions. Therefore,

$$q_{i}=\frac{D(C_{i}^{i}-C_{i}^{0})}{N_{v}}\times 10^{9}$$ (Eq 3)

where C^i^_i_ is the concentration of i in the inlet in (mmol/L), C^i^_o_ is the concentration of i in the outlet in (mmol/L), N_v_ is the concentration of viable cells in (10^6^ cells/ml), and D is the dilution rate of the culture in (1/h).

In transient states, the concentration of each metabolite varies with time. Therefore,

$$\frac{dC_{i}}{dt}=DC_{i}^{i}-DC_{i}^{0}-q_{i}N_{v}10^{3}$$ (Eq 4)

In this case, qi was calculated numerically, first by fitting a function that describes the variation in metabolite concentration over time *C* ^*0*^_i_ (t). Next, this function was derived and subsequently evaluated.

### 2.6. Calculation of the accumulation rate constant and half-accumulation time of the intracellular protein

The data were normalised to the value of the parameter at the beginning of the perturbations, allowing a comparison of the relative changes over the time; in addition, the data were log transformed. Protein accumulation was determined using the accumulation rate constant (k), by employing the accumulation curves described in Eq 5.

$$P=P_{0}e^{kt}$$ (Eq 5)

Where P is the intracellular protein at time t, and P_0_ is the intracellular protein at initial time t_0_. *k* is the accumulation rate constant.

Subsequently, the protein half-accumulation time (t_1/2_) was calculated from the accumulation rate constant (Eq 6).

$$t_{1/2}=\frac{LN(2)}{k}$$ (Eq 6)

### 2.7 Statistical Analysis

All cultures under each condition were performed in duplicate, and two independent samples were taken at each time point for every culture, with analytical measurements carried out separately. Values are expressed as the mean standard error. Analysis of variance for factorial designs of two or three factors was used to compare all results by Design-Expert® 7 for Windows. Additionally, Tukey’s test was performed to compare the different results obtained for each experimental design. Statistical significance was considered for p<0.05.

## Results and Discussion

### 3.1. Evaluation of the ERAD I and ERAD II pathway activities over rht-PA: Cell growth and protein processing

The importance of the ERAD I and ERAD II pathways over post-translational events of rht-PA was investigated in batch cultures of CHO cells at 37°C and 33°C. Tunicamycin (TM), an inhibitor of protein glycosylation, was used to inhibit protein folding and consequently forced the degradation of the protein by any available ERAD pathway. Tunicamycin has been shown to effectively inhibit protein glycosylation without affecting protein synthesis [28,29]. Inhibitors of ERAD I or ERAD II pathways were used in combination with TM to block protein degradation, and then the variation of the intracellular rht-PA quantity was observed. Previously, the concentration of each inhibitor was selected so that the cellular viability did not decrease below 90% after 28 hours of culture.

**Fig 1:**
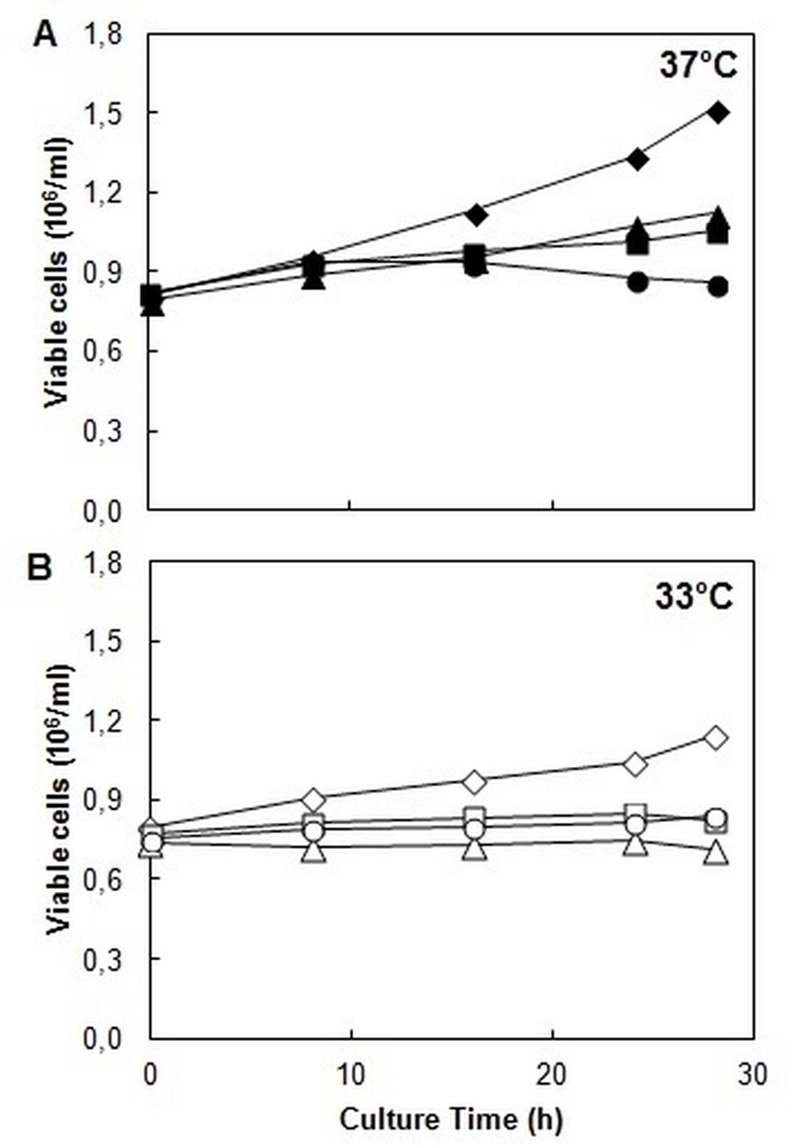
CHO cell growth profile in batch culture at different temperatures and under glycosylation/ERAD inhibition. **A:** Viable cell concentration at 37°C. **B:** Viable cell concentration at 33°C. ◆ Control culture; ■ Tunicamicyn culture; ▲ Tunicamicyn/ERAD-I culture; ● Tunicamicyn/ERAD-II culture. ◇ Control culture; □ Tunicamicyn culture; △ Tunicamicyn/ERAD-I culture; ○ Tunicamicyn/ERAD-II culture.

Fig 1 shows profiles of cell growth at 37°C and 33°C in the presence of the inhibitors. The control culture reached a maximum cell concentration of 1.5 x 10^6^ cells/ml at 37°C, whereas the control culture at 33°C, at the same time of culture at 37°C reached 1.1 x 10^6^ cell/ml, as expected due to the lower culture temperature and the concomitant lower growth kinetic. The specific cell growth rate was 0.024 h^-1^ at 37°C and 0.018 h^-1^ at 33°C. Treatment with the inhibitor caused cell growth to decrease at 37°C and 33°C, exhibited a more prominent effect at 37°C. However, in all cases, viability was greater than 95% (data not shown).

**Fig 2:**
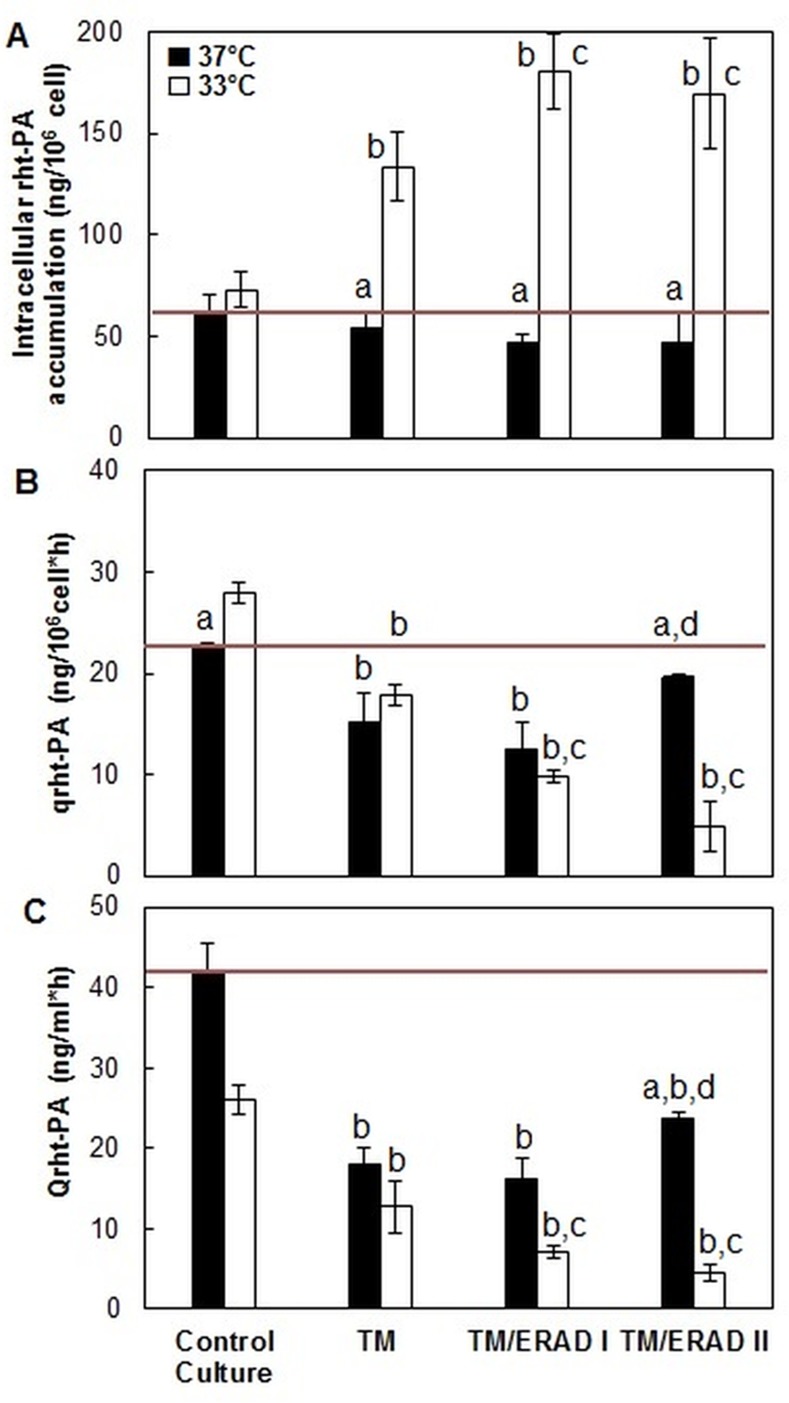
Intracellular accumulation, specific and volumetric productivity of rht-PA at different culture temperatures and under glycosylation/ERAD inhibition, in batch cultures. ANOVA and the Tukey’s test. Statistical significance was considered for p<0.05. ^a^: Significant differences respect to counterpart culture at different T°; ^b^: Significant differences respect to CC; ^c^: Significant differences respect to TM; ^d^: Significant differences respect to TM/ERAD I.

Depending on the culture temperature used, treatment with various inhibitors caused changes in the concentration of rht-PA(i) (Fig 2). Batch cultures carried out at 37°C showed a slight reduction in the concentration of rht-PA(i), as a result of the possible blocking of the glycosylation and ERAD pathways. Moreover, the batch cultures performed at 33°C exhibited an increase in the concentration of rht-PA(i) by 40%, 101% and 76% most likely due to the inhibition of the glycosylation process and glycosylation/ERAD I and glycosylation/ERAD II pathways, respectively. In this case, the accumulation of rht-PA(i) was adjusted to first-order kinetics, from which accumulation constant values and the average accumulation time were calculated, for each experimental condition (Table 1).

**Table 1:** Intracellular rht-PA half-accumulation time and accumulation constants under mild hypothermia and glycosylation/ERAD inhibition in the batch culture.

	33°C	
	k (1/h)	t1/2 (h)
**CC**	0.0007	990
**TM**	0.0088^a^	79a
**TM/ERAD I**	0.0223^a^ ^,^ ^b^	31^a^ ^,^ ^b^
**TM/ERAD II**	0.0186^a^ ^,^ ^b^	37^a^ ^,^ ^b^

CC: Control Culture; TM: Tunicamycin Culture; TM/ERAD I: Tunicamycin/ERAD I Culture; TM/ERAD II: Tunicamycin/ERAD II Culture.

Statistical significance was considered for p<0.05

^a^: Significant differences respect to CC, Tukey´s test.

^b^: Significant differences respect to TM, Tukey´s test.

The data clearly show that tunicamycin treatment at 33°C induced an increase in intracellular rht-PA, in agreement with an expected decrease in rht-PA glycosylation together with a lower rate of rht-PA secretion from the cells. Furthermore, ERAD I and II inhibition leads to an increased intracellular rht-PA and a decreased rate of rht-PA secretion, consistent with an important contribution by ERAD I and II to ER rht-PA degradation pathways at 33°C. Instead, at 37°C, no intracellular rht-PA accumulation was observed after treatment with TM or ERAD I or II inhibitors and only a slight decrease in rht-PA secretion (Fig 2). Since it is unlikely that the pharmacological properties of the chemicals used could be drastically decreasing at 4°C higher temperature, Thus, our data suggest that a non-ERAD degradation could be present at 37°C in these cells (e.g., Mancini et al., 2003; [30]; Donoso et al., 2005; [31]; Shenkman et al., 2007; [32]). Alternatively, a combined effect of rht-PA synthesis inhibition could be induced by TM in CHO cells at 37°C (e.g., Shenkman et al., 2007; [32]; Chen et al., 2015; [33]; So et al., 2015; [34]) but not at 33°C, together with a possible non-ERAD intracellular rht-PA degradation at 33°C.

Inhibiting glycosylation and either the ERAD I or II pathway caused a larger increase of the intracellular quantity of r-protein, revealing the participation of these pathways in protein degradation, with average accumulation times of 34 h (Table 1). The activities of the ERAD I and ERAD II pathways have been reported for different proteins expressed by CHO cells [35,30] and other cell lines [36,17], revealing their participation and possible influence on the final result of the protein synthesis process. However, the actions of these pathways under the condition of mild hypothermia have not been evaluated previously.

Even though there were no significant differences in the control rht-PA cell content at 37°C and 33°C in batch cultures, this last condition presented a significant increase in the rht-PA specific productivity that suggest an increase in synthesis [37] together with a likely increased processing of the r-protein [11] under mild hypothermia condition.

### 3.2. Effect of the specific growth rate and mild hypothermia on ERAD over rht-PA under inhibitory conditions

To assess separately the effects of specific cell growth rate and mild hypothermia on the degradation of rht-PA(i) by ERAD pathways, chemostat cultures of CHO cells were carried out at two dilution rates, High-D (0.017 h^-1^) and Low-D (0.012 h^-1^), and at two culture temperatures (37°C and 33°C). Once SS had been reached and the cells were maintained in a physiological state defined by the variables established, the system was perturbed by inhibiting the ERAD I and II ERAD pathways to determine the variation in the quantity of rht-PA(i), as a result of the blockage of protein degradation. At SS, the concentration of rht-PA(i) was significantly different in the four initial conditions evaluated (Table 2). At High-D, the perturbation of the system caused no significant change in the intracellular protein quantity, under all temperature conditions evaluated. However, at Low-D, the increase in the intracellular protein concentration was significantly higher for both temperature conditions (Table 2). The observed accumulation of rht-PA(i) by blocking the ERAD I and II pathways showed average accumulation times of 84 h at 37°C and 323 h at 33°C (Table 3). Notably, at Low-D the condition of mild hypothermia caused a lower degree of accumulation of the intracellular protein in comparison to 37°C. Thus, the average accumulation time was decreased by approximately 75% under ERAD I and ERAD II inhibition (Table 3). This indicates a positive effect of mild hypothermia on the processing of the endoplasmic reticulum.

**Table 2:** Intracellular rht-PA accumulation at different temperatures and ERAD inhibition in the chemostat culture.

		rht-PA(i) (ng/10^6^cell)	% of intracellular rht-PA(i) after 48 h of treatment **	
Dilution rate (h^-1^)	Temperature (°C)	At steady state (SS)	Under ERAD-I blockeage	Under ERAD-II blockeage
**0.017**	37	7.9 ± 1.1	120 ± 3.7	107 ± 0.4
	33	6.5 ± 0.6^c^	117 ± 0.3	115 ± 1.0
**0.012**	37	4.7 ± 1.1^b^	185 ± 4.1^a^ ^,^ ^b^	242 ± 16.9^a^ ^,^ ^b^
	33	6.1 ± 0.5^c^	139 ± 1.4^a^ ^,^ ^b^ ^,^ ^c^	150 ± 5.1^a^ ^,^ ^b^ ^,^ ^c^

**Respective SS.

Statistical significance was considered for p<0.05

^a^: Significant differences respect to SS, Tukey´s test.

^b^: Significant differences respect to counterpart culture at same T° and different D, Tukey´s test

^c^: Significant differences respect to counterpart culture at same Treatment and different T°, Tukey´s test

**Table 3:** Intracellular rht-PA half-accumulation time and accumulation constants at different temperatures and ERAD inhibition in the chemostat culture.

	0.012 (1/h)				0.017 (1/h)			
	37°C		33°C		37°C		33°C	
	k (1/h)	t1/2 (h)	k (1/h)	t1/2 (h)	k (1/h)	t1/2 (h)	k (1/h)	t1/2 (h)
**ERAD I**	0.0082^a^ ^,^ ^b^	84^a^ ^,^ ^b^	0.0021^a^ ^,^ ^b^	330^a^ ^,^ ^b^	0.0004	1732	0.0004	1732
**ERAD II**	0.0083^a^ ^,^ ^b^	83^a^ ^,^ ^b^	0.0022^a^ ^,^ ^b^	315^a^ ^,^ ^b^	0.0002	3465	0.0006	1588

Statistical significance was considered for p<0.05

^a^: Significant differences respect to counterpart culture at same T° and different D, Tukey´s test

^b^: Significant differences respect to counterpart culture at same Treatment and different T°, Tukey´s test

Regardless of the temperature conditions used, the behaviour of the cultures at Low-D suggests that the accumulation of intracellular protein based on the ERAD I and II pathways and as observed in batch culture is related to the decrease in specific growth rate of the cells rather than to the mild hypothermia condition itself. However, the lower degree of accumulation of intracellular protein at 33°C and the low dilution rate indicates a positive effect of mild hypothermia on the folding capacity of the endoplasmic reticulum in comparison to the 37°C condition. This result was obtained by decreasing the amount of unfolded protein subjected to degradation at the lower temperature condition.

The effect of mild hypothermia on the protein folding capacity has been exclusively reported for batch cultures and has shown improved post-translational processing, increased ER size and increased ER chaperone expression [11,12,13]. However, these apparent improvements are the result of the combined effects of reducing both the temperature and specific growth rate.

To our knowledge, there exists no extensive information regarding the effect of specific cell growth rate on the processing capacity on CHO cell culture. This work suggests that independent of the temperature conditions, the effect of specific growth rate would be relevant and significant with respect to protein synthesis, as it is for the central metabolism [20].

### 3.4. Analysis of cell behaviour by PCA: Effect of specific cell growth rate and mild hypothermia condition

To provide a better understanding of the influence of the variables studied, the cell behaviour of the chemostat cultures was analysed with a multivariate approach. The variables modelled were biomass, specific consumption and productivity of nutrients and metabolic by-products, specific productivity and enzymatic activity of extracellular rht-PA, and intracellular quantity of rht-PA. The observations were obtained from cell cultures in SS tested at 48 and 72 hours post-treatment with ERAD I inhibitor, with ERAD II inhibitor and without inhibitor.

**Fig 3:**
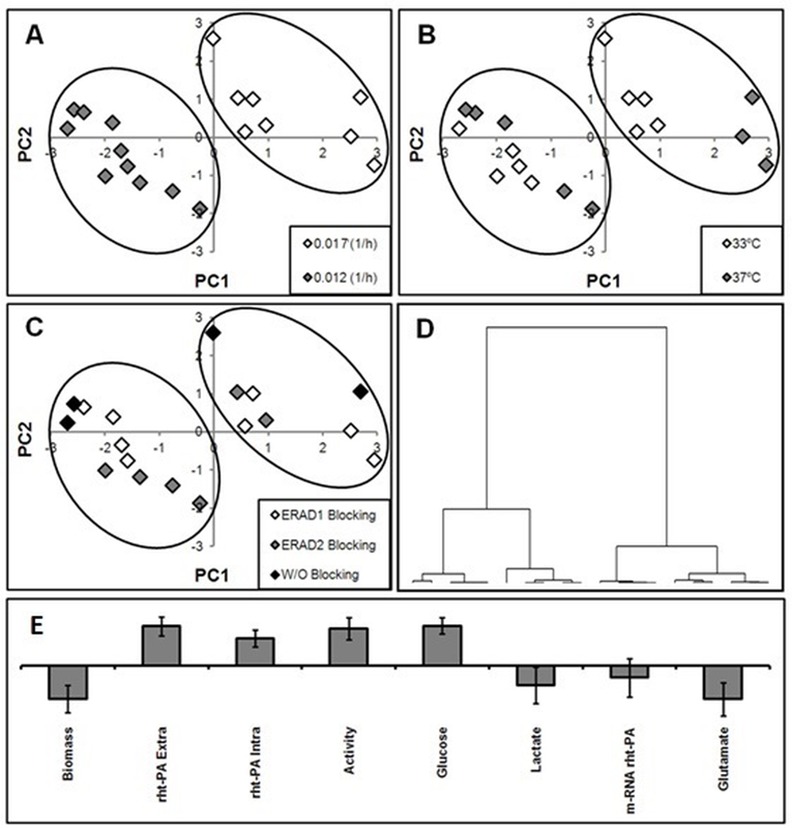
Principal Component Analysis (PCA). A, B and C: Hyperplane between the first and second principal components (PC1 and PC2, respectively). A, B and C show the same hyperplane, but the symbols are coloured differently in each inset to indicate the factors studied. D: Dendrogram (Ward distance) of the observation used in PCA to identify the two defined groups in A, B and C. E: Loadings of the first principal component (PC1) shown in A, B or C (bars represent C.I. 95%).

Fig 3 shows the results of a PCA, where the PC1 and PC2 hyperplanes explained 71.7% of the variability (Fig 3A, 3B and 3C). The hyperplane showed that the data can be arranged into two defined groups, indicating the presence of two cell behaviours. This latter idea was confirmed using a clustering analysis (Fig 3D), which indicated the presence of two separate clusters with a dissimilarity of approximately 63%. Both defined clusters are associated with different dilution rates (specific growth rates) but not with temperatures or ERAD-blocking, demonstrating that, in terms of the behaviour of the cells, the increased variability is explained by the specific rate at which cell population grows.

Because a good discrimination of the dilution rates into two clusters was performed by PC1, the variables significantly associated with PC1 were studied to explain this phenomenon. The PC1-loadings (Fig 3E) showed that the increase of rht-PA(i) content and the reduction of biomass are strongly associated with cell behaviour. Moreover, the PC1-loadings exhibited an inverse relationship between biomass and serine protease activity, suggesting that low processing capacity, which is associated with the lowest growth rate, may affect the enzymatic activity of the recombinant protein.

## Conclusion

In this work, it was observed that the rht-PA protein is susceptible to degradation by both ERAD pathways studied, as verified by the use of glycosylation and ERAD pathway inhibitors in batch culture. The effect of the mild hypothermia, on these pathways revealed that processing of rht-PA is sensitive to this condition in batch culture. The effect of separating the culture temperature from the cell growth rate was verified using chemostat culture as an experimental approach. This revealed that ERAD pathways are more sensitive to a reduction of the specific cell growth rate than to low temperature. In contrast, a reduction in temperature may positively affect protein processing by reducing the mis/unfolded r-protein destined for degradation. Moreover, PCA indicated that the integrated performance displayed by the CHO cells was modulated predominantly by their specific cell growth rate, indicating that culture temperature as an operational variable has a lower weighted effect than the specific growth rate, in the range of the conditions evaluated in this work.
